# Hydronephrosis Due to Intraureteral Migration of Missed Intrauterine Device

**DOI:** 10.7759/cureus.53820

**Published:** 2024-02-08

**Authors:** Kadir Karkin, Ediz Vuruşkan, Mubariz Aydamirov, Eyüp Kaplan, Bugra Aksay, Güçlü Gürlen

**Affiliations:** 1 Department of Urology, Health Sciences University, Adana City Training and Research Hospital, Adana, TUR; 2 Department of Urology, Başkent University, Alanya Application and Research Center, Antalya, TUR; 3 Department of Urology, Abdulkadir Yüksel State Hospital, Gaziantep, TUR

**Keywords:** double-j stent, ureteric obstruction, hydronephrosis, migration, intrauterine device

## Abstract

Migration of intrauterine devices (IUDs) to the ureter causing ureteral obstruction is an extremely rare event. A 45-year-old female patient was admitted to our hospital with a complaint of pain in the right flank and inferior right quadrant of the abdomen. On genitourinary system ultrasonography, grade 3 hydronephrosis was found in the right kidney. On the abdominopelvic radiography, there was an appearance consistent with two IUDs, one in the region consistent with the course of the right distal ureter and the other in the usual localisation. The first IUD was thought to have spontaneously detached from the uterus, so a second IUD was implanted. A right-sided extravesical ureteroneocystostomy (Lich-Gregoir) operation was performed. The operation was terminated with the placement of a 4.8 French, 26 cm double-J stent in the ureter. The patient was followed up in our hospital for 26 months and she was asymptomatic during follow-up, hydronephrosis was resolved completely, and no complications were encountered during follow-up clinical and ultrasonography examinations.

## Introduction

An intrauterine device (IUD) is an extremely effective and reversible form of contraception that is durable in the long term [1]. Intrauterine contraception is widespread worldwide and 13.9% of the 1.16 billion females aged 15-49 years prefer this method [2]. Although IUD is a safe and effective method, it is known that there can be some complications in their use. In particular, perforation of the uterus with the IUD during placement can result in migration of the IUD to the peritoneum, colon, or bladder [1]. In the literature, the bladder was reported to be the most frequent site of migration [3]. Migration of IUD to the ureter causing ureteral obstruction is an extremely rare event.

In this case report, the complications occurring in the urinary system caused by an IUD migrating to the ureter lumen and treatment of these complications are presented and discussed in light of current literature.

## Case presentation

**Figure 1:**
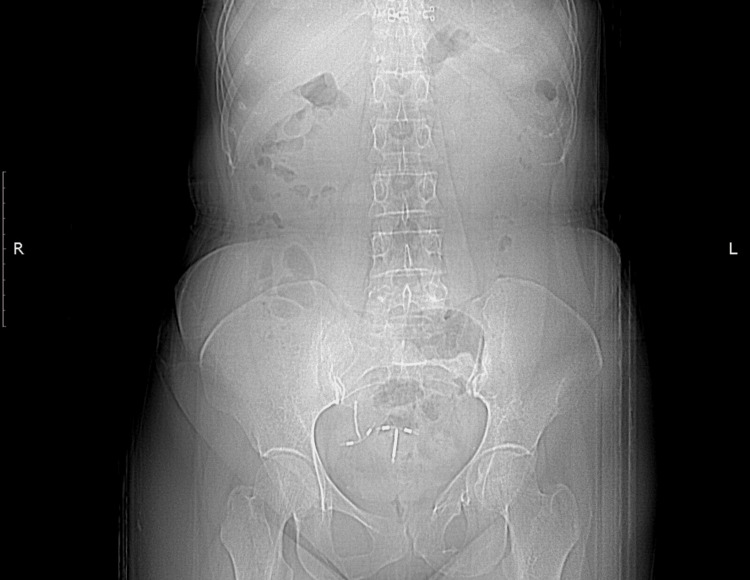
On abdominopelvic X-ray, there was an appearance consistent with two intrauterine devices, one in the region consistent with the course of the right distal ureter and the other in the normal localisation.

**Figure 2:**
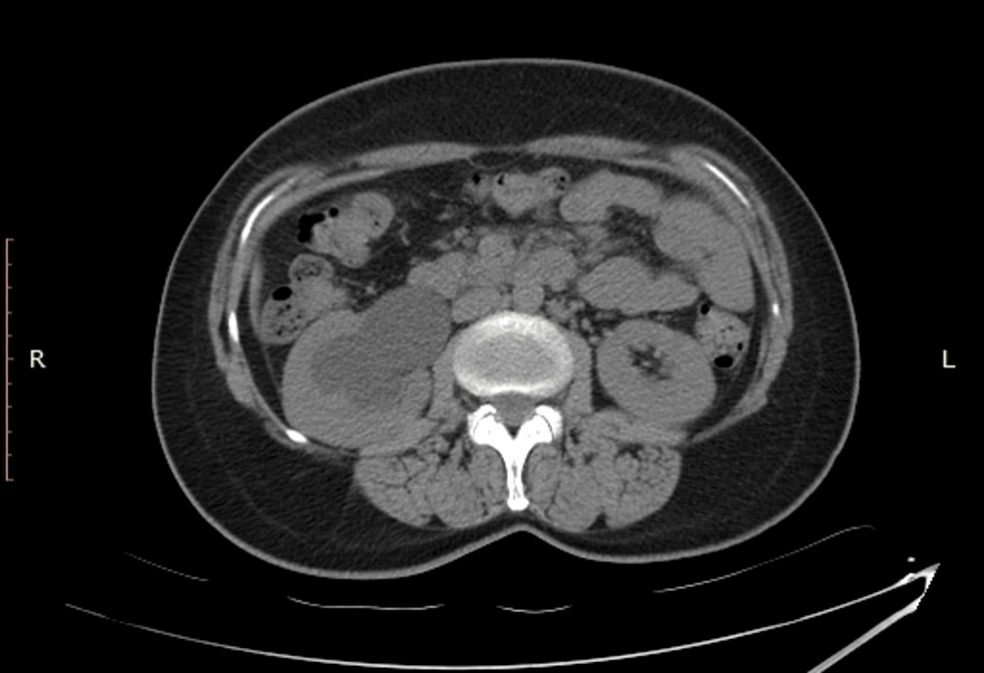
Right grade 3 hydronephrosis due to invading the ureteral wall of the migrated intrauterine device.

**Figure 3:**
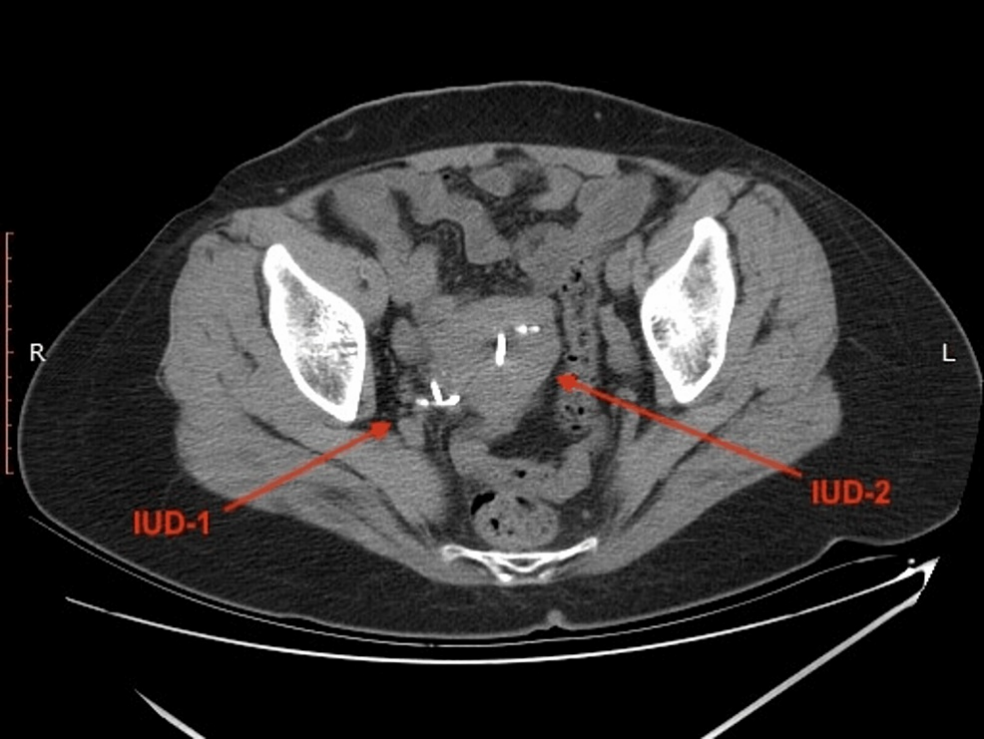
On CT examination, IUD-1 was observed in the distal ureter and IUD-2 was seen in the uterus. IUD: intrauterine device.

**Figure 4:**
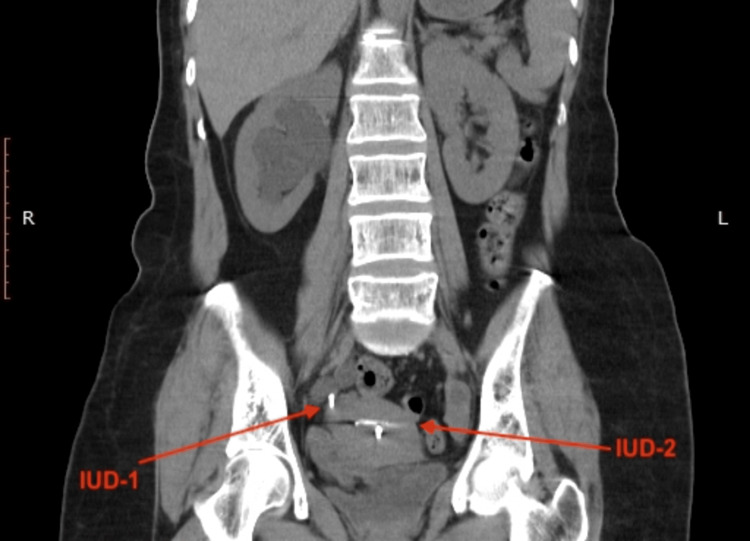
Right hydronephrosis and both IUDs were visible in the coronal planes of computed tomography. IUD: intrauterine device.

**Figure 5:**
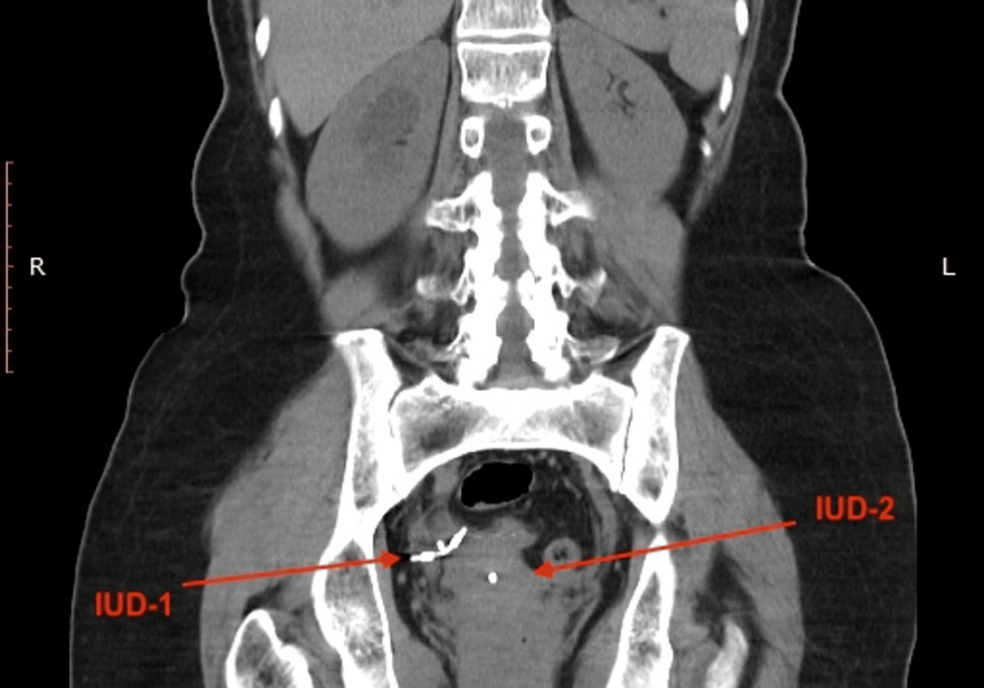
Right hydronephrosis and both IUDs were visible in the coronal planes of computed tomography. IUD: intrauterine device.

A 45-year-old female patient was admitted several times to gynaecology and general surgery clinics with complaints of pain in the right flank and inferior right quadrant of the abdomen. Despite different medical treatments, the complaints have not subsided. On the last presentation at the general surgery clinic with the same complaints, right-side hydronephrosis was detected on abdominal ultrasonography (USG) and the patient was referred to the urology clinic. During the urological evaluation, it was learned from medical history that a contraceptive IUD was implanted in 2004 by the gynaecology department and the patient attended regular gynaecological follow-up examinations until 2010. During a gynaecological examination and transvaginal USG in 2010, the IUD could not be seen in the uterus and with the thought that it had spontaneously dropped, a new IUD was implanted. Physical examination found the patient felt tenderness in the right flank. The urine test results were completely normal. On the urinary system USG, grade 3 hydronephrosis was observed in the right kidney. On abdominopelvic X-ray, there was an appearance consistent with two IUDs; one in the region probably compatible with the course of the right distal ureter and the other in the normal localisation (Figure 1). From these findings, it was speculated that the first IUD could have migrated, and to fully confirm the exact localisation, non-contrast computed tomography (CT) imaging was performed. On the CT examination, a foreign body (the first IUD) was observed in the distal ureter. It had invaded the ureteral wall and grade 3 hydroureteronephrosis was observed to probably be associated with obstruction (Figures 2-5). Diagnostic ureteroscopy was planned for the patient under general anaesthesia.

**Figure 6:**
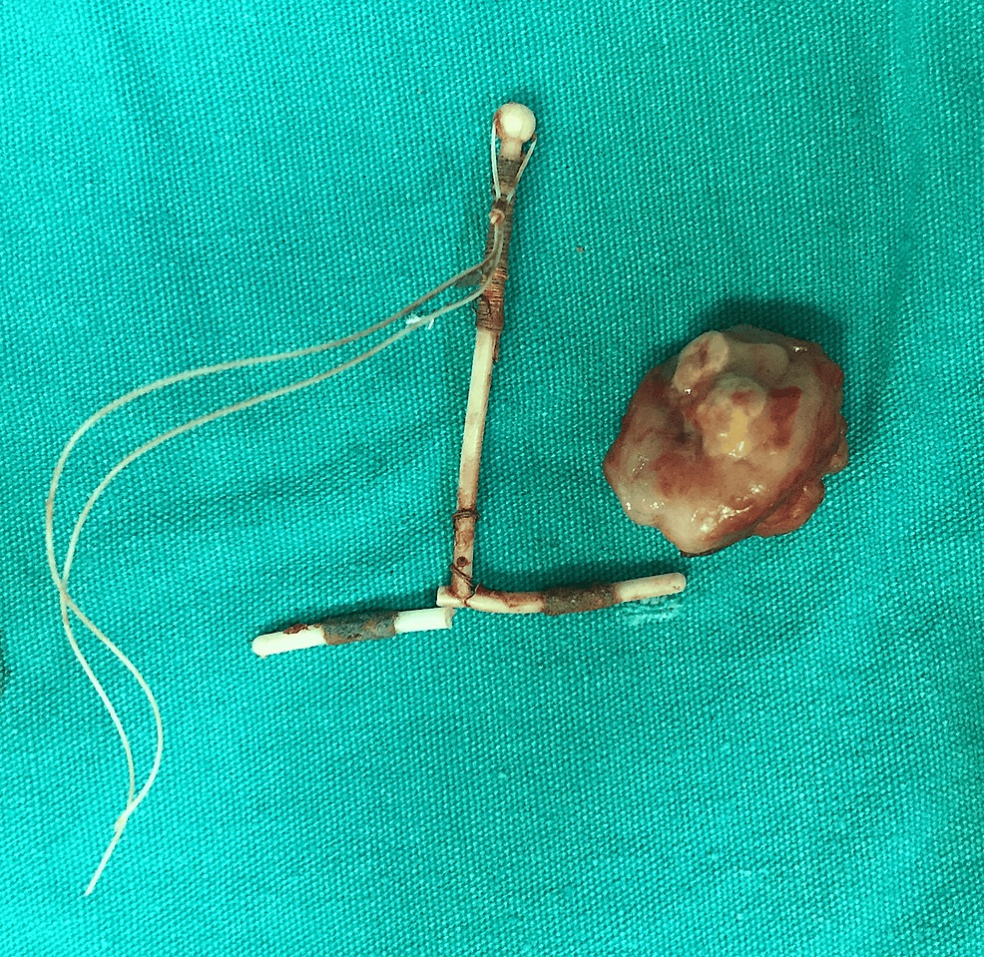
The intrauterine device was excised and removed.

The patient was informed about the operation and a signed informed consent form was obtained. A negative urine culture was obtained before the procedure and the patient was admitted for surgery. Right-sided ureteroscopy was applied and the distal ureter was observed to be completely obstructed by the IUD. A right-side Gibson incision was made and the ureter was reached, then by freeing the ureter, the IUD was excised and removed (Figure 6). The migrated IUD was observed to have caused obstruction and severe fibrosis was detected in the ureter and surrounding tissue. A right-side extravesical ureteroneocystostomy (Lich-Gregoir) operation was performed. The operation was terminated with the placement of a 4.8 French, 26 cm double-J (DJ) stent in the ureter. The patient was discharged from the hospital on postoperative day four.

**Figure 7:**
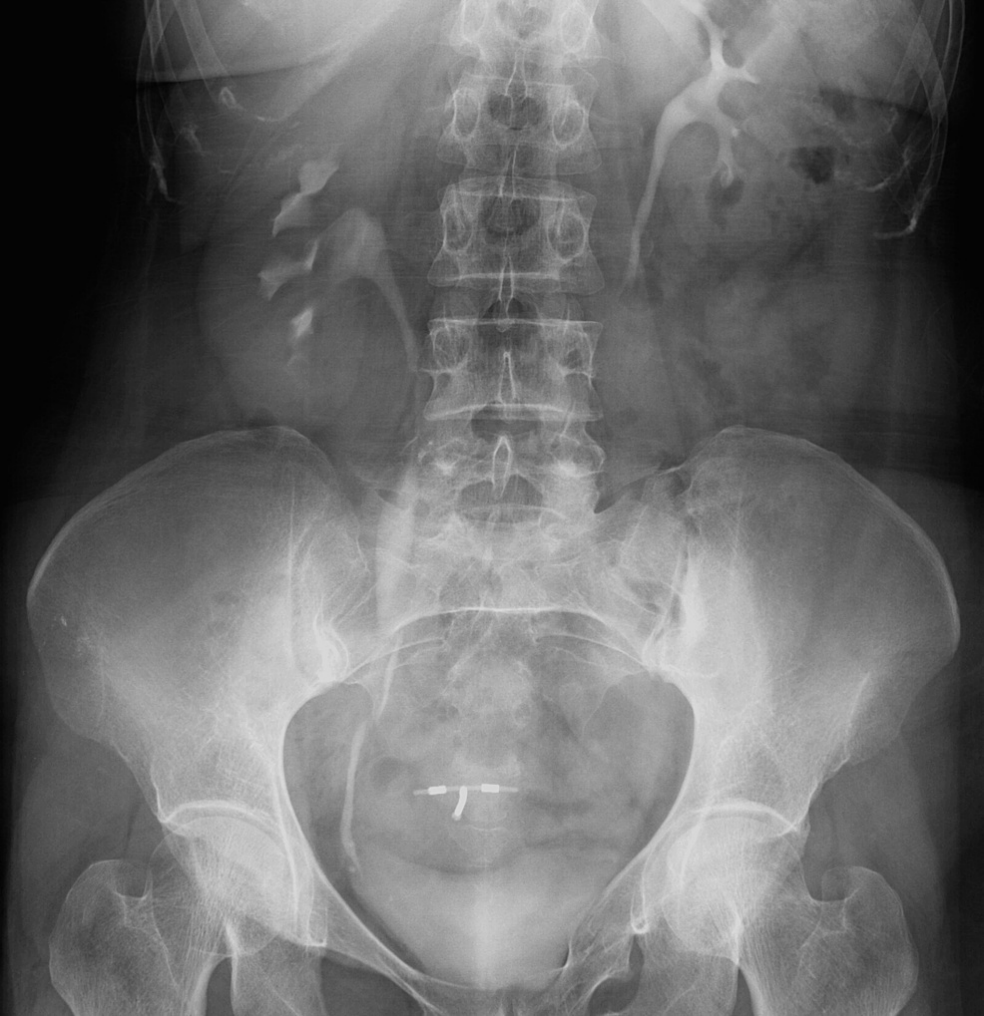
On intravenous urography performed nine months later, the right-side hydronephrosis was seen to have disappeared and the patient had no complaints of pain.

The DJ stent was removed three months postoperatively. On intravenous urography (IVU), which was performed nine months later, right-sided hydronephrosis was observed to have disappeared and the patient had no complaints of pain (Figure 7). The patient was followed in our clinic for 26 months and no complications were encountered during follow-up clinical and ultrasonographic examinations.

## Discussion

Although a rarely encountered phenomenon, uterine perforation by IUD is a significant clinical problem, which should be explained to the patient and requires diagnosis and appropriate treatment. Most cases are due to traumatic perforation during placement of the device. In addition, secondary perforation can occur with gradual erosion. Partial perforation may become complete perforation in the long term. In large-scale studies, the incidence of IUD-related uterus perforation was reported as 0.4-1.6 per 1000 placements [1,4]. Possible risk factors for perforation include inexperience of the clinician, lactation, postpartum insertion (less than six months postpartum), low parity, and high number of abortus [1,4,5]. Following perforation, there may be migration of the IUD to the colon, small intestine, appendix, or rectum [1]. Although migration to the urinary system is uncommon, migrations to the ureter and even to the urethra were reported in the literature [6,7]. An IUD can cause bladder stone formation, vesicouterine fistula, and fibrosis in the pelvic ureter [6].

Although uterine perforation is a potentially serious complication, it may be asymptomatic, and even in some cases, the IUD may not be identified until months or years after insertion [4,8]. However, a migrated IUD can cause severe comorbidities, such as pelvic abscess, intestinal obstruction and perforation, and even death [6]. In the current case, the patient was admitted with complaints of flank pain 12 years after placement of the IUD.

In addition to being very uncommon, another uniqueness of this presented case is that the IUD could not be found in the uterine cavity six years after placement and as it was thought to have spontaneously fallen, a second IUD was inserted. Diagnosis of perforation and migration can be made by gynaecological examination, USG, and abdominopelvic X-ray, but with CT, more accurate information is obtained about the exact localisation of the IUD, abdominal migration, and the relationship with adjacent tissues and organs [3]. The inability to find the lost IUD at that time can be attributed to insufficient evaluation with the use of ultrasound only and it should also be kept in mind that ultrasonic evaluation is more successful for determination of the intrauterine localisation of IUDs rather than extrauterine IUDs. Previous studies reported that ultrasonographic scanning to visualise perforating IUDs is unsuccessful in more than half of patients [1,9]. As IUDs are radio-opaque, they can be detected easily on abdominopelvic X-ray films. If the simple test of X-ray imaging had been used in addition to the usage of ultrasound imaging for this particular patient, the complications associated with the migrated IUD may not have developed, or it could have been removed laparoscopically without the need for an operation with higher rates of morbidity, such as ureteroneocystostomy.

Despite the discussion related to the necessity to remove an inert extrauterine IUD, the general consensus is removal as it could cause an inflammatory reaction. However, a migrated IUD that caused complications must definitely be removed [6,10]. Endoscopic removal of IUD after complete intravesical migration is a safe, feasible, and minimally invasive method [6] but endoscopic removal is not possible in partial migrations to the bladder and ureteral wall. An IUD that has migrated to the ureter can cause foreign body inflammation, fibrosis, and even ischaemic necrosis [3]. In a study by Yang et al., an IUD that migrated to the ureter was laparoscopically removed and a DJ stent was inserted, but as hydronephrosis continued during follow-up, ureteroneocystostomy was applied [3]. Especially, with the development of obstruction and severe fibrosis in the ureter in cases such as the patient reported here, excision of the fibrotic ureter segment together with the IUD and ureteroneocystostomy seems to be the safest option in terms of preventing the development of ureteral stricture in the future.

## Conclusions

Although an IUD is a safe, effective, and reversible contraceptive method, migration to unusual locations may be seen together with uterine perforation. Therefore, in patients with a history of IUD placement, migration to the ureter should be kept in mind for differential diagnosis as a very rare cause of hydronephrosis. The IUD should be removed surgically and these patients must be followed up in the long term for the potential risk of ureteral stricture. In addition, patients using an IUD for contraceptive purposes must be followed up regularly to ensure that the IUD remains in the proper position. Additionally, for the investigation of a lost IUD, ultrasonographic findings alone are not reliable and simple tests such as abdominopelvic X-ray should not be neglected.
